# *Psidium guajava* Seed Oil Reduces the Severity of Colitis Induced by Dextran Sulfate Sodium by Modulating the Intestinal Microbiota and Restoring the Intestinal Barrier

**DOI:** 10.3390/foods13172668

**Published:** 2024-08-24

**Authors:** Hanwen Zhang, Guoxin Shen, Hongling Lu, Chenkai Jiang, Wenjun Hu, Qihong Jiang, Xingwei Xiang, Zongxing Wang, Lin Chen

**Affiliations:** 1 College of Food Science and Technology, Zhejiang University of Technology, Hangzhou 310014, China; 221122260077@zjut.edu.cn (H.Z.); xxw11086@126.com (X.X.); 2 Zhejiang Academy of Agricultural Sciences, Hangzhou 310021, Chinagreenbreezekai@126.com (C.J.); guyuexingshi@163.com (W.H.);; 3 Zhejiang Forestry Technology Extended Station, Hangzhou 311300, China

**Keywords:** *Psidium guajava* seed oil, linolenic acid, intestinal microbiota, colitis

## Abstract

The oil derived from *Psidium guajava* seeds (TKSO) exhibits an abundance of diverse unsaturated fatty acids, notably oleic, linoleic, and α-linolenic acids, conferring substantial health advantages in addressing metabolic irregularities and human diseases. This research endeavor focused on elucidating the impacts of TKSO on colonic inflammatory responses and intestinal microbiota alterations in a murine model of colitis induced by dextran sulfate sodium (DSS), demonstrated that substantial supplementation with TKSO reduces the severity of colitis induced by DSS. Furthermore, TKSO effectively attenuated the abundance and expression of proinflammatory mediators while augmenting the expression of tight junction proteins in DSS-challenged mice. Beyond this, TKSO intervention modulated the intestinal microbial composition in DSS-induced colitis mice, specifically by enhancing the relative presence of *Lactobacillus*, *Norank_f_Muribaculaceae*, and *Lachnospiraceae_NK4A136_*group, while concurrently diminishing the abundance of *Turicibacter*. Additionally, an analysis of short-chain fatty acids (SCFAs) revealed noteworthy elevations in acetic, propionic, isobutyric, and butyric acids, and total SCFAs levels in TKSO-treated mice. In summary, these findings underscore the potential of TKSO to reduce the severity of colitis induced by DSS in mice through intricate modulation of the intestinal microbiota, metabolite profiles, and intestinal barrier repair, thereby presenting a promising avenue for the development of therapeutic strategies against intestinal inflammatory conditions.

## 1. Introduction

Ulcerative colitis (UC), an inflammatory bowel disease (IBD) variant, is experiencing a surge in incidence within our country [1]. The initial manifestations of UC vary widely, with bloody diarrhea being the primary early indicator [2]. Additional symptomatic presentations encompass abdominal distress, hematochezia, weight loss, and vomiting [3]. In the absence of timely intervention, the likelihood of developing colon cancer significantly intensifies [4]. Therapeutic options for UC include 5-aminosalicylic acid derivatives, corticosteroids, and immunosuppressive agents. However, these interventions are often accompanied by a myriad of adverse effects, including headaches, nausea, vomiting, abdominal discomfort, and dermatological rashes, with no guarantee of preventing disease recurrence [5]. Consequently, the exploration of efficacious dietary supplements, devoid of or exhibiting minimal adverse reactions, holds great promise as a strategy for mitigating and/or managing UC. Notably, dietary lipids are increasingly acknowledged as crucial modulators of intestinal disease predisposition, emphasizing their importance among nutritional factors [6,7,8].

The medicinal foodstuff, *Psidium guajava*, which originates from the Juniperus communis plant, boasts a lengthy cultivation history in China [9]. It is renowned for its lung-moistening, phlegm-dispersing, anti-inflammatory, analgesic, and intestinal-lubricating properties, aiding in bowel movements [10]. TKSO, an oil derived from *Psidium guajava*, surpasses traditional oilseed crops, like rapeseed and soybean, in terms of oil content, exceeding 35% [11]. This oil is enriched with unsaturated fatty acids, comprising over 60% of its total fat composition [12], featuring oleic acid alongside conjugated linoleic and conjugated linolenic (triureidoic) acids [13]. Notably, conjugated linoleic acid exhibits salutary physiological effects, encompassing cancer prevention, weight management, immune system fortification, and blood lipid moderation [14,15]. Its counterpart, conjugated linolenic acid, elicits toxicity towards human tumor cells, inhibiting carcinogenesis and garnering considerable attention due to its potent properties [16]. Furthermore, the intake of conjugated linolenic acid promotes lipid metabolism and contributes to a reduction in blood lipids [17].

Recently, more and more studies have revealed the UC therapeutic activity of some natural oils [18,19], such as olive oil [20], perilla oil [18], garlic oil [21], flaxseed oil [6], walnut oil [22], and emu oil [23]. The anti-IBD activities of these natural oils from different plants and animals have been validated in a large number of experimental UC animal models and some clinical trials [24]. However, the interventional effect of *Psidium guajava* seed oil on colitis in mice is not clear. In the present study, we used a mouse model of dextran sodium sulfate (DSS)-induced colitis to investigate the mechanisms of the hypolipidemic, antioxidant, and anti-inflammatory effects of *Psidium guajava* seed oil intervention in mice with colitis. This is to provide theoretical support for the development of *Psidium guajava* seed oil as an oil for the human diet or as a functional food for the prevention of colitis.

## 2. Materials and Methods

### 2.1. Materials and Reagents

**Figure 1:**
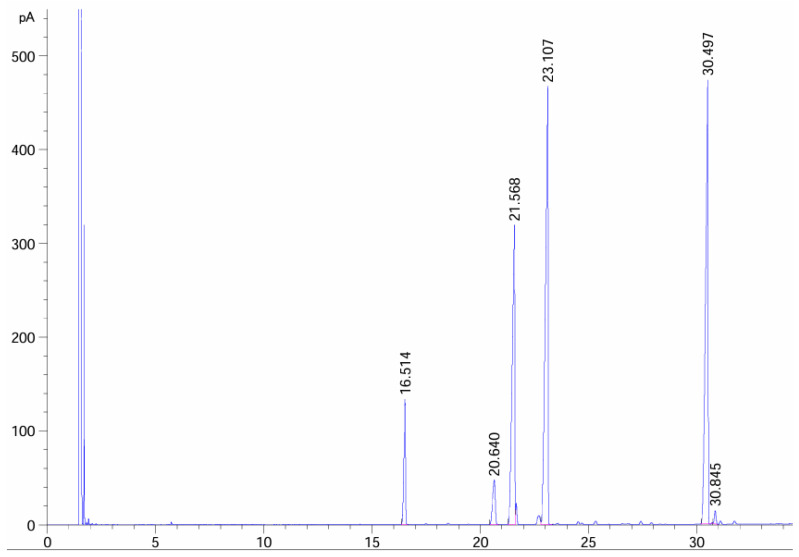
Through gas chromatography analysis, we determined the composition of TKSO.

*Psidium guajava* seed oil (TKSO), prepared and provided by the Sericulture and Tea Research Institute, Zhejiang Academy of Agricultural Sciences, was used to determine the composition of TKSO by the GC method, as shown in Figure 1 and Table 1. Determination of TKSO composition by gas chromatography indicated that linoleic acids, oleic acids, and linolenic acids constituted 87.386% of the total fatty acid content. ELISA test kits were purchased from Wuhan Doctoral Bio-engineering Co. Ltd. (Wuhan, China). RNA extraction kits, reverse transcription kits, fluorescence quantification kits, and four primary antibodies were purchased from Beijing Tiangen Co. Ltd. in Beijing, China. Dextran sulfate sodium salt (DSS, Mv: 36–50 kDa) was purchased from MP Biomedicals, Santa Ana, CA, USA. Plain mouse food and mouse bedding were obtained from Shuangshi Laboratory Animal Feed Technology Co. in Suzhou, China. All other chemical reagents used were of analytical grade.

### 2.2. Experimental Methods

**Figure 2:**
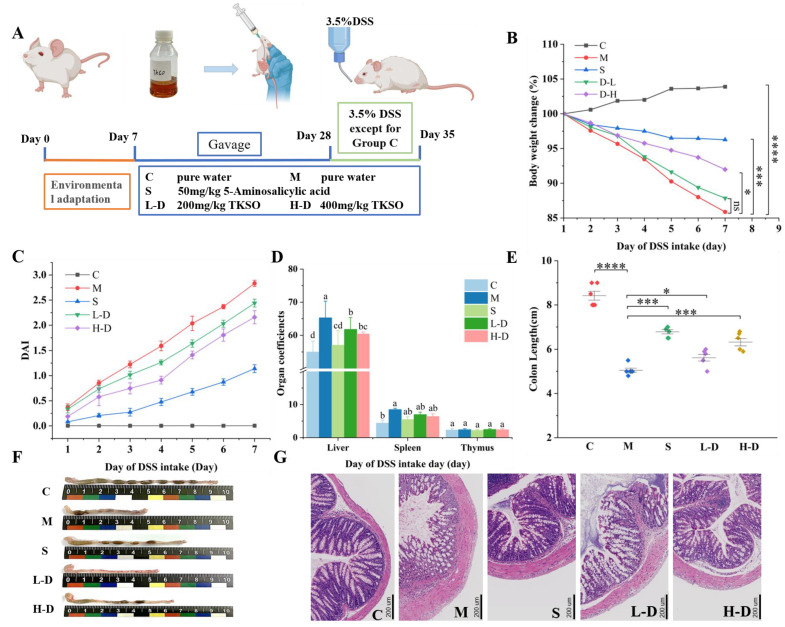
(**A**) Design of the experimental protocol for mice studies; (**B**) weight changes in each group of mice; (**C**) DAI (disease activity index) scores in each group of mice; (**D**) the effect of TKSO on the organ index in colitis mice; (**E**) the colon tissue length of each group of mice; (**F**) the apparent characteristics of the colon tissue in each group; (**G**) effect of TKSO on the structure of colonic tissue in mice with colitis. Microscopic images of a H&E-stained mice colonic tissue representative section; bar’s length is 200 um. * *p* < 0.05, *** *p* < 0.001, **** *p* < 0.0001. Data marked with different letters in each column represent significant differences (*p* < 0.05).

Animal experiments were conducted in accordance with the Chinese Standard for Laboratory Animals and the Regulations on the Use and Care of Laboratory Animals by the Ethics Committee of Zhejiang Polytechnic University (License No. SYXK (Zhe) 2020-0022). Thirty male BALB/c mice weighing 16–18 g and aged 4 weeks were used in the experiments. The mice were housed at a temperature of 23 ± 2 °C and a relative humidity of 60 ± 5%, with a light/dark cycle of 12 h/12 h, and they had access to food and water ad libitum. After a week of acclimatization feeding, the mice were randomly assigned to five groups, from the 7th day to the 28th day. Group C were administered pure water by gavage; Group M were administered pure water by gavage; Group S received 50 mg/kg of 5-aminosalicylic acid per day by gavage; Group L-D received 200 mg/kg/d of TKSO by gavage; and Group H-D received 400 mg/kg/d of TKSO by gavage. According to the recommended daily oil intake of the Human Diet Pyramid, the daily oil consumption in the mice receiving 400 mg/kg/d was equivalent to 16.2 mg/kg/d adults in humans (body weight 60 kg) and 24 mg/kg/d in children (body weight 20 kg), which is significantly lower than the daily dose (25–30 g/day) recommended by the Chinese Nutrition Society [25]. The drinking water for each group was pure water. From the 28th to the 35th day, except for the control group, the other groups of mice were continuously administered with drinking water containing DSS (3.5% *v*/*w*) for one week to induce experimental ulcerative colitis. The gavage method remained the same as during days 7–28. The daily amount of DSS consumed by each group of mice was recorded, and it was found that there was no significant difference in drinking volume among the groups [26]. Please refer to Supplementary Figure S1. The experimental plan for each group of mice is shown in Figure 2A.

#### 2.2.1. Recording of Body Weight, Disease Activity Index, and Organ Indexes

From day 28 to day 35 of the experiment, the body weight of mice in each group was recorded daily, and the percentage decrease in weight relative to day 28 was calculated. At the end of the experiment, mice were treated under anesthesia. After blood was taken from the mouse eye socket, the mice were decapitated and executed. Thymus, spleen, and liver were carefully collected, rinsed clean with PBS buffer, and then drained with filter paper, weighed sequentially, and recorded. The organ index was calculated for each organ according to the following formula:

Organ index = (organ weight mg/mouse body weight g) × 10

The colon tissues were collected, and their lengths were measured. In a sterilized environment, the mouse cecum was cut to collect its contents in sterile test tubes and stored at −80 °C for intestinal microbiota analysis.

#### 2.2.2. Morphologic Observation of Colon Tissue

For ultrastructural observation, fresh colon tissues were first divided into small pieces and transferred to 2.5% glutaraldehyde fixative pre-cooled to 4 °C for fixation, followed by different treatments. Scanning electron microscopy sample processing: After the colon tissue blocks were fixed again with 1% osmium tetroxide solution for 1 h, they were dehydrated using a gradient ethanol solution and pure acetone solution and then dried at the liquid CO_2_ critical point. Subsequently, the samples were glued on a sample stage, coated with thin gold using an ion sputter coater, and transferred to an electron microscope to obtain images for observation of the colon tissue surface. Transmission electron microscopy sample processing: Similarly fixed and dehydrated colon tissue blocks were embedded using Spurr resin overnight, then cut into thin slices for uranyl acetate–lead citrate double-staining and transferred to a transmission electron microscope to obtain images for observation of the colon tissue surface.

A portion of the colon was fixed in 4% paraformaldehyde for preservation. The well-preserved intestinal tissues were dehydrated using graded concentrations of anhydrous ethanol, followed by clearing, paraffin embedding, and cutting into approximately 4 μm sections. After decolorization and deparaffinization, the sections were stained with hematoxylin–eosin (H&E). The stained sections were then observed under a light microscope, and photographs were taken. Liver tissue injury was assessed by examining the stained sections after dehydration, clearing, and sealing treatments.

#### 2.2.3. Measurement of Serum Biochemical Indicator

After collection, blood samples were centrifuged at 12,000 rpm for 10 min at 4 °C. The supernatant was then carefully removed, and the serum was assayed for levels of the inflammatory factors interleukin 6 (IL-6), tumor necrosis factor (TNF-α), and interferon (INF-β) using ELISA kits according to the manufacturer’s instructions.

#### 2.2.4. Determination of mRNA Expression Levels of Related Factors in Colonic Tissues

RNA was extracted from colon tissues using an RNA extraction kit according to the manufacturer’s instructions. cDNA was then synthesized using a reverse transcription kit according to the manufacturer’s instructions. cDNA samples were stored at −80 °C until further use. Primers were designed based on the sequences obtained from the NCBI and synthesized by Shanghai Sangong Bioengineering Co., Ltd. (Shanghai, China). The primer sequences used in this study are shown in Table 2.

The cDNA samples were diluted to the same concentration and quantified using a fluorescence-based assay. The PCR reaction consisted of an initial denaturation step at 95 °C for 30 s, followed by 40 cycles at 95 °C for 5 s and 34 s at the appropriate annealing temperature. For details on the annealing temperatures, please refer to Supplementary Table S1. The expression level of the target gene was normalized to the expression of β-actin as an internal reference gene. The relative expression level of the target gene was calculated using the 2^−ΔΔCt^ method.

#### 2.2.5. Determination of Short-Chain Fatty Acids in the Cecum

To extract metabolites from the appendix, 20–100 mg of appendix content was dissolved in 1 mL of pure water. Then, 100 μL of 50 μg/mL isocaproic acid was added and the mixture was shaken for 2 min. The solution was centrifuged at 4 °C, 12,000 rpm for 1 min, and the supernatant was collected. The supernatant was passed through a 0.05 μm aqueous filter membrane into a new tube. We added 90 μL of 50% sulfuric acid and 600 μL of ether to the new tube. The solution was shaken for 20–30 s and then centrifuged at 12,000 rpm for 5 min. Analytes were analyzed using gas chromatography–mass spectrometry (GC-MS). The chromatographic conditions used were a DB-WAX quartz capillary column (30 m × 0.25 mm, 0.25 μm) with a temperature increase starting at 90 °C for 3 min, followed by a temperature increase of 10 °C/min to 120 °C, 5 °C/min to 150 °C, and 25 °C/min to 250 °C, and held at 250 °C for 5 min. The carrier gas used was helium at a flow rate of 1.0 mL/min. The injection volume was 10 μL, the inlet temperature was 250 °C, and the detector temperature was 230 °C.

#### 2.2.6. 16S rRNA Sequencing of the Mouse Gut Microbiome

Using the MOBIO PowerSoil^®^ DNA Extraction Kit (MOBIO, Carlsbad, CA, USA), total genomic DNA from the mouse fecal contents was extracted to obtain the gut microbiome. The quality of the DNA was verified by running it on a 1% agarose gel, and it was appropriately diluted with sterile water to reach the same concentration. The diluted genomic DNA was then used as a template to amplify the highly variable V3 to V4 region of the 16S rRNA using the specific primers 338F (5′-ACTCCTACGGGAGGCAGCA-3′) and 806R (5′-GGACTACHVGGGTWTCTAAT-3′) from Shanghai Meiheng Biopharmaceutical Technology Co., Ltd. (Shanghai, China). The PCR reaction was carried out using TransGen’s TransStart Fastpfu DNA polymerase and a high-efficiency, high-fidelity enzyme. Afterward, the PCR products were evaluated by agarose gel electrophoresis at a 2% concentration. An Illumina library was constructed using the TruSeq™ DNA Sample Preparation Kit, and the library was identified and sequenced using the Illumina MiSeq PE300 platform (San Diego, CA, USA).

The quality of the obtained forward and reverse sequences was optimized using FLASH software (version 1.2.11). Sequences with 97% similarity were grouped into multiple chimera-free operational taxonomic units (OTUs) using Meijibio Bioconfidence Cloud. Microbial community composition was analyzed for each sample using Mothur software (version 1.30.2), and alpha diversity was analyzed using USEARCH software (version 7.0.1090). To compare beta diversity between groups based on sample distance, principal coordinate analysis (PCoA) was performed using QIIME software (version 1.9.1) and R software (version 3.3.1). Significant differences in microbial community structure between groups at the phylum and genus levels were analyzed using Metastats 7.0 software. Correlation heat map analysis was performed to determine the relationship between gut flora, fecal metabolites, and biochemical indicators.

#### 2.2.7. Statistical Analysis

One-way analysis of variance (ANOVA) using SPSS version 25.0 was employed to analyze the results of the physical and chemical tests. Post hoc multiple comparisons between groups were performed using the Duncan method. Origin 2024 software was used for data visualization. Experimental data are expressed as mean ± standard deviation (SD), with *p* < 0.05 or *p* < 0.01 considered statistically significant.

## 3. Results and Discussion

### 3.1. Effect of Psidium guajava Seed Oil on Body Weight and DAI Scores of Colitis Mice

The percentage change in the body weight of the mice is shown in Figure 2B. The mice in group C, fed a normal diet, were mobile, had shiny fur, and their body weights remained on an upward trend. After the addition of DSS (3.5% *v*/*w*) to the drinking water, the mice in groups M, S, L-D, and H-D showed different degrees of body weight loss on day 3. They exhibited symptoms, such as blood in the stool, diarrhea, reduced dietary activity, and deterioration of fur, leading to a significant increase in the DAI score (Figure 2C). These observations suggested that DSS successfully induced colitis in the mice. Notably, the S, L-D, and H-D groups showed moderated weight loss (*p* < 0.05) compared with the M group, with some improvement in diet and activity. The DAI scores were lower in these groups than in the DSS model group, with the most pronounced effects observed in the H-D group. We also analyzed the weekly weight changes in the mice from the beginning of gavage to the end of modeling. Please refer to Supplementary Figure S2. It can be seen that before the mice consumed DSS, the weight of each group of mice showed an upward trend. After drinking DSS, the weight of the mice significantly decreased, with the M group showing the most pronounced decrease. This also indicates that the modeling was successful. This study has a limitation in that it did not measure the extent of colitis induced by initial DSS, which might have led to an imprecise assessment of our treatment efficacy. This omission could affect the accuracy of our conclusions. In future related studies, we will concentrate on accurately measuring the severity of colitis induced by initial DSS to gather more comprehensive data.

### 3.2. Effect of Psidium guajava Seed Oil on Colonic Organ Index in Colitis Mice

The results of the organ weights and counts for each group of mice are shown in Figure 2D. Compared with group C, the liver and spleen indices of the mice were significantly higher after DSS induction, while the thymus index was significantly decreased (*p* < 0.05). This suggests that DSS induction caused stress in the immune organs of the mice. TKSO treatment alleviated the increase in liver and spleen indices and mitigated the decrease in the thymus index to some extent, showing a significant difference from the DSS group (*p* < 0.05).

### 3.3. Effect of Psidium guajava Seed Oil on the Morphology of Colon Tissue in Mice with Colitis

As shown in Figure 2F, the colonic tissues of mice in group C appeared flesh pink in color, had greater overall length compared to other experimental groups, and contained completely formed feces. In contrast, DSS induction significantly shortened the length of colon tissue (*p* < 0.05), resulting in thinner tissue with unformed stools visible in the intestinal lumen. Additionally, atrophy, congestion of the cecum, and partial dark red coloration of the colonic surface were observed. Administration of 5-aminosalicylic acid and TKSO treatment reduced the shortening of the colon due to DSS, decreased bleeding in the intestinal lumen, and overall improved bowel condition. The L-D group showed a more significant difference (*p* < 0.05) compared to the H-D group, which also showed a significant difference (*p* < 0.05) compared to the DSS group. Figure 2E indicates that the colon length in the L-D and H-D groups was closer to that of group C compared to group M. This suggests that TKSO reduces the severity of colitis induced by DSS.

### 3.4. Effects of Psidium guajava Seed Oil on the Colonic Tissue Structure of Mice with Colitis

The colonic tissue sections of mice were magnified and observed (Figure 2G). The intestinal epithelial cells and crypt structures of mice in group C were intact, the glands were neatly arranged, and there were no ulcers or inflammatory cell infiltration. In contrast, DSS-induced intestinal structures appeared damaged, with portions of the intestinal wall necrotic, crypt structures and goblet cells distorted, abnormal gland structures, and submucosal inflammatory cell infiltration. These symptoms indicated severe damage to the colonic tissues of DSS-induced mice. Both L-D and H-D treatments improved the intestinal damage caused by DSS, with tissue ulceration reduced and the basic structure restored, although some ulcers were still observed in certain areas. The H-D treatment was more effective in maintaining the structural integrity of the colon and reducing inflammation.

### 3.5. Effects of Psidium guajava Seed Oil on the Ultrastructure of Colon in Colitis Mice

**Figure 3:**
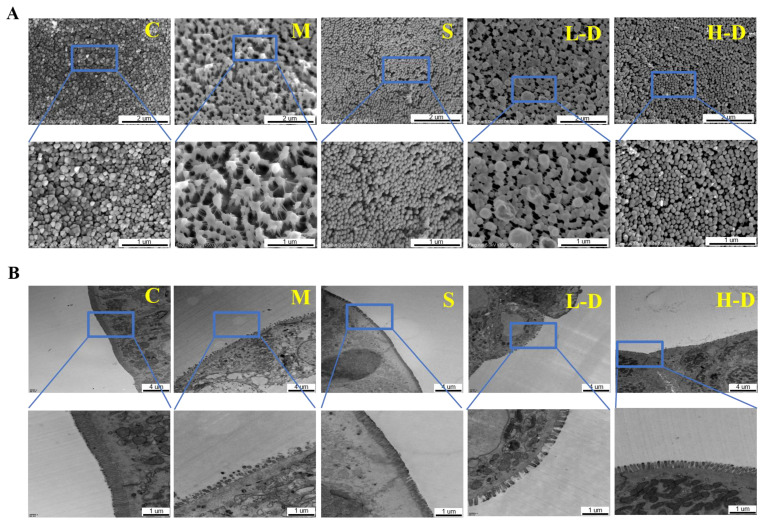
Effect of TKSO on the structure of colonic tissue in mice with colitis. (**A**) The surface of colonic epithelium was observed under SEM; bar’s length is 2 μm or 1 μm. (**B**) The surface of colonic epithelium was observed under TEM; bar’s length is 4 μm or 1 μm.

To further understand the protective effect of *Psidium guajava* seed oil on DSS-induced intestinal injury, ultrastructural observation of the colonic epithelium was carried out using SEM and TEM (Figure 3A,B). The colonic epithelium in group C displayed well-arranged and densely packed microvilli of uniform shape and size, with clear expression of the tight junctions. The surface of the intestinal tract was smooth and free of collapsing adhesions or foreign matter. In group M, the microvilli of the colonic epithelium became sparse and shortened, with a disorganized arrangement and disrupted tight junctions. In group S, the microvillous structure of the colonic epithelium was reduced compared to group C, but it was tighter and more organized than in group M, with very few foreign bodies on the surface. Groups L-D and H-D showed a reduction in overall microvillous structural pathology, with a smoother arrangement and increased visibility of the tight junctions. Additionally, microvilli regained surface smoothness, and collapsed atrophy was reduced. However, the L-D group was not as effective as the H-D group.

### 3.6. Effect of Psidium guajava Seed Oil on Inflammatory Factors in Serum and Colonic Tissues of Mice

**Figure 4:**
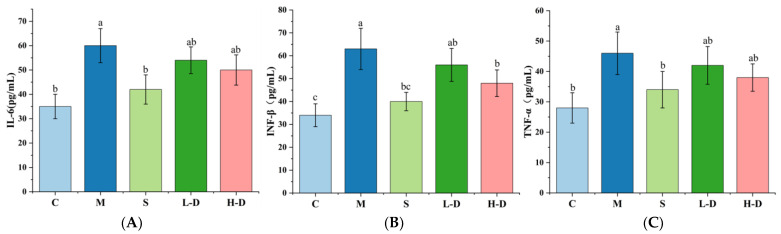
Effects of TKSO on inflammatory cytokines in the serum of mice. (**A**) The levels of IL-6 in serum. (**B**) The levels of INF-β in serum. (**C**) The levels of TNF-α in serum. Data marked with different letters in each column represent significant differences (*p* < 0.05).

Inflammatory factors are crucial signaling molecules for inflammatory responses in vivo, and their expression levels are positively correlated with the severity of UC. The results are shown in Figure 4A–C. Comparing group M with group C revealed that the expression levels of IL-6, INF-β, and TNF-α in the serum and colon of mice were significantly increased *(p *< 0.05), indicating that DSS led to an increase in inflammatory factors in the colon tissue. Different doses of TKSO treatment reduced the expression of inflammatory factors IL-6, TNF-α, and INF-β. Among them, the results after H-D treatment were closer to the control group (*p* > 0.05). This suggests that the intake of TKSO is beneficial in reducing the level of inflammatory factors in vivo, thereby alleviating the inflammatory response in UC mice.

### 3.7. Effect of Psidium guajava Seed Oil on the Expression Level of Colon Related mRNA Genes

**Figure 5:**
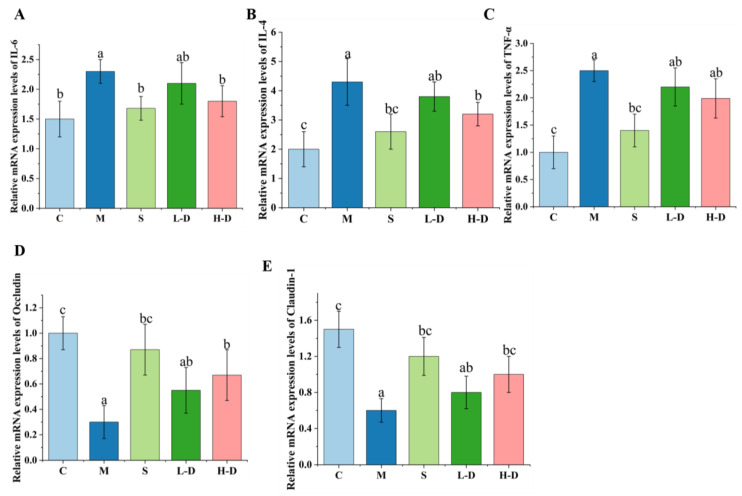
Effect of TKSO on the expression level of related mRNA gene in mice colon tissue: (**A**) IL-6, (**B**) IL-4, (**C**) TNF-α, (**D**) occludin, and (**E**) claudin-1. Different letters indicate significant differences (*p* < 0.05).

The expression levels of relevant mRNA genes in the colon tissues of mice in each group were detected using RT-PCR. As shown in Figure 5A–C, similar to the results of the cytokine assay, the mRNA expression levels of TNF-α, IL-6, and INF-β in the colon tissues of mice in group M were significantly increased (*p* < 0.05). After H-D treatment, the mRNA expression levels of TNF-α, IL-6, and INF-β in the colon tissues were reduced and approached normal levels.

In addition, Figure 5D,E show that the tight junction proteins occludin and claudin-1 were abundantly expressed in the colonic tissues of mice in the normal group in a healthy state. The expression levels of these two tight junction proteins in the colonic tissues of mice with DSS-induced colitis were significantly lower compared to those in group C (*p* < 0.05), indicating that the intestinal barrier of the mice was damaged. The experimental results demonstrated that the relative expression of the genes occludin and claudin-1 could be improved by different doses of TKSO treatment in a dose-dependent manner. Both the H-D and L-D groups showed a significant increase compared to the DSS group (*p* < 0.05).

### 3.8. Effect of Guarana Acid Oil on the Composition of Intestinal Microbiota of Mice with Colitis

#### 3.8.1. Visualization of TKSO Distribution in Fecal Samples of Colitis Mice

**Figure 6:**
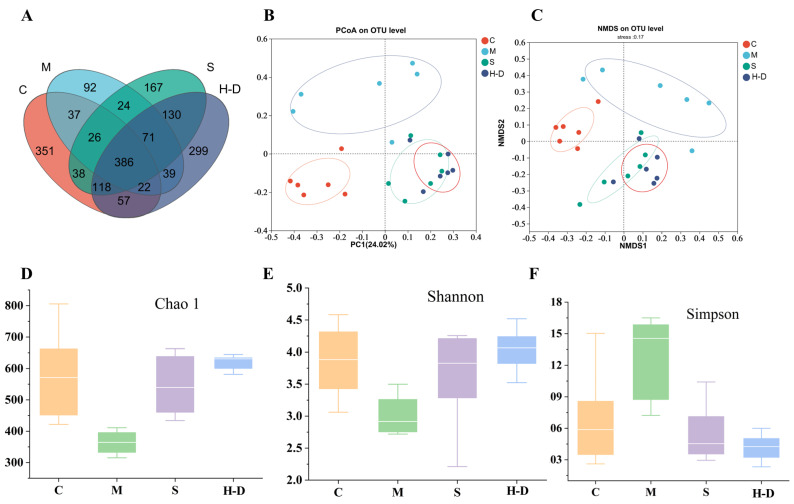
Effect of TKSO on the structure and diversity of intestinal flora in mice. (**A**) Venn diagram, (**B**) PCoA analysis diagram, (**C**) NMDS analysis diagram, (**D**) Chao1 index, (**E**) Shannon index, and (**F**) Simpson index.

It has been reported that the occurrence of IBD is closely related to the imbalance of intestinal microbiota, and a reduction in intestinal microbiota diversity is an important characteristic of IBD patients [27]. We sequenced and analyzed the H-D group, which had the best results as described above, along with the C, M, and S groups. After high-throughput sequencing based on the Illumina MiSeq sequencing platform, a total of 1857 OTUs were obtained from all the samples of the cecum contents of the mice (Figure 6A). The C group contained 1035 OTUs packaged in the intestinal microbiota; the M group contained 697 OTUs; the S group contained 934 OTUs; and the H-D group contained 1122 OTUs. A further comparison of the OTUs between the groups showed that the C group had 351 unique OTUs, whereas the M group had only 92 unique OTUs, with an overlap of only 37 OTUs between the 2 groups. However, the number of OTUs in both the S and H-D groups increased to varying degrees compared to the M group, with 38 and 57 OTUs overlapping with the C group, respectively. Compared with group M, group H-D had more identical OTUs with group C. This suggests that TKSO treatment facilitates the normalization of the intestinal microbiota in mice with colitis after DSS induction.

#### 3.8.2. Effect of TKSO on Diversity in Colony Mice

The diversity index is a composite measure of richness and evenness within a particular region or ecosystem [28]. The Chao index is used to estimate the number of OTUs in a community using the Chao1 algorithm and is commonly used to indicate community richness [29]. The Simpson index describes the degree of evenness in the distribution of species within a community, with higher index values indicating greater diversity of the community. In the DSS-treated samples (Figure 6D–F), the Chao and Shannon indices decreased, while the Simpson index increased. TKSO treatment increased the Chao and Shannon indices, suggesting that TKSO helps to improve the homogeneity and relative abundance of gut flora. Mouse gut microbiota diversity was assessed using principal coordinate analysis (PCoA); this is a dimensionality reduction technique used to project high-dimensional data into a low-dimensional space to facilitate data visualization and analysis, as shown in Figure 6B. Each point in the figure represents a sample from the same group with the same color, and the distance between two points reflects the difference between the two samples. The results showed that the samples from groups C and M had relatively large distances. The relative concentrations and overall dispersion of the samples from these two groups indicated a significant difference in overall community composition between groups C and M. Treatment with the positive control drug 5-aminosalicylic acid resulted in a shift in the gut microbiota composition of mice toward that of group C. On the other hand, TKSO administration led to a significant change in the gut microbiota composition of mice, manifested by a shortening of the distance between groups H-D and C at the PC1 level. Additionally, non-metric multidimensional scaling (NMDS) is a dimensionality reduction method used to explore the similarities or differences between samples, based on the distances or dissimilarities between the samples. Figure 6C yielded similar results to the PCoA analysis. These results suggest that TKSO can alter the diversity of the gut microbiota in mice.

#### 3.8.3. Composition and Abundance Analysis at the Gate Level

**Figure 7:**
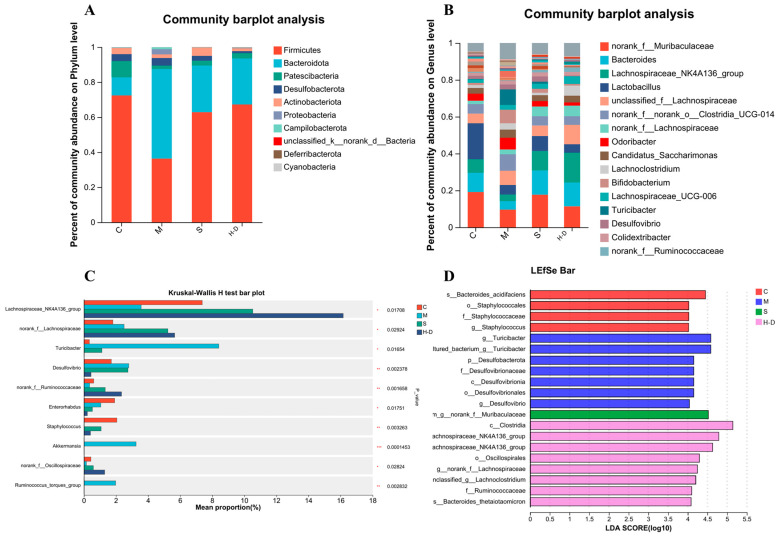
Effect of TKSO on intestinal microbiota composition in NAFLD mice. (**A**) Phylum level community abundance, (**B**) genus level community abundance, (**C**) genus level Kruskal–Wallis rank sum test, (**D**) and genus level LEfSe bar test.

To further assess the effects of TKSO on the intestinal microbiota, we analyzed the community abundance at the phylum level. As shown in Figure 7A, the first six phyla included Firmicutes, Bacteroidetes, Proteobacteria, Desulfovibrionaceae, Actinobacteria, and Verrucomicrobia, with Firmicutes, Bacteroidetes, and Proteobacteria being the three dominant phyla. The average proportions of Firmicutes, Bacteroidetes, and Proteobacteria in group C mice were 72.4%, 10.2%, and 9.3%, respectively. In group M mice, the mean proportions of these three phyla were 36.3%, 51.1%, and 1.9%, respectively. The mean proportion of Bacteroidetes was significantly higher (*p* < 0.01) and the mean proportion of Firmicutes was significantly lower (*p* < 0.01) in group M compared with group C mice. Additionally, the Firmicutes/Bacteroidetes (F/B) ratio was decreased, suggesting a significant imbalance of the intestinal microbiota after DSS induction (*p* < 0.01). A previous study noted that increasing the F/B ratio is beneficial for increasing intestinal short-chain fatty acid production, decreasing the risk of infection, and promoting weight gain [30].

#### 3.8.4. Composition and Abundance Analysis at the Genus Level

At the genus level, we selected the 16 most abundant genera for comparison and analyzed compositional differences among them (Figure 7B). Among the four tested groups, the genera *norank_f_Muribaculaceae*, *Mycobacterium*, *Lachnospiraceae_NK4A136_group*, and *Lactobacillus* were the dominant genera. The abundance of *Lactobacillus*, *norank_f_Muribaculaceae*, Bacteroidetes, and *Lachnospiraceae_NK4A136_group* was significantly lower in group M mice compared to group C mice (*p* < 0.01). After TKSO treatment, the abundance of these genera significantly increased (*p* < 0.01), aligning with the trend observed at the phylum level. *Lactobacillus* are known to inhibit the growth of spoilage and pathogenic bacteria in the intestine [31], reduce blood ammonia and cholesterol levels, and maintain the balance of intestinal microbiota. They also have immunomodulatory effects [32], such as promoting cell division, antibody production, macrophage activation, and inducing the production of interferon, thereby enhancing the body’s disease-fighting capabilities. *Norank_f_Muribaculaceae* has been reported to be positively associated with the expression of barrier function genes in newborn piglets [33]. Another SCFA-producing bacterium, *Lachnospiraceae_NK4A136_group*, has been negatively associated with inflammation [34]. Consistent with previous findings, TKSO treatment significantly increased the abundance of *Lactobacillus* and *Lachnospiraceae_NK4A136_group* (Figure 7C). In addition to these genera, *Turicibacter*, *Staphylococcus*, *Enterococcus*, and *Romboutsia* also exhibited varying levels of change. *Turicibacter* and *Romboutsia* were significantly increased, while *Staphylococcus* and *Enterococcus* were significantly decreased in the model group compared to the control group. However, these changes were reversed after TKSO treatment (*p* = 0.002, *p* = 0.015, *p* = 0.012, and *p* = 0.004, respectively). *Turicibacter* has been shown to facilitate colorectal cancer (CRC) development by promoting chronic inflammation, DNA damage, and the generation of biologically active oncogenic metabolites [35]. Reference [36] demonstrated that 2′-fucosyl lactose (2′FL) and oligogalactose (GOS) attenuated DSS-induced colitis in mice while inhibiting *Romboutsia*, consistent with our findings. As shown in Figure 7D, we also analyzed the prominent taxa in each group based on LDA-LEfSe analysis. It was observed that group C was enriched with Staphylococcus, group M was enriched with *Turicibacter*, group S was enriched with *Norank_f_Muribaculaceae*, and group H-D was enriched with *Lachnospiraceae_NK4A136_group*. These results are consistent with our previous studies, indicating that TKSO has a regulatory effect on the intestinal microbiota of DSS-treated mice.

#### 3.8.5. Correlation Analysis of Short-Chain Fatty Acids, Colon Biochemical Indicators and Intestinal Microbiota 

**Figure 8:**
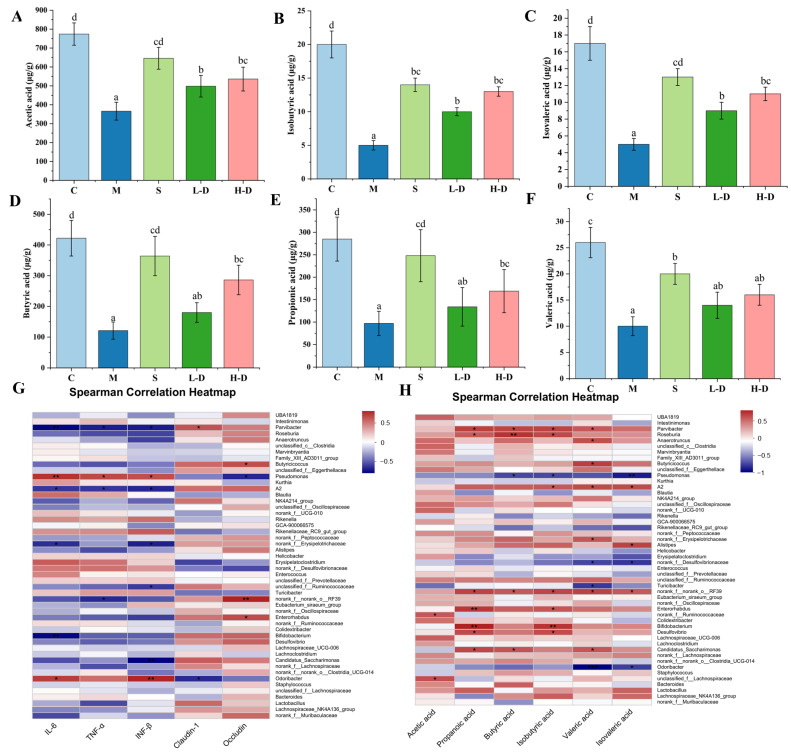
Correlation analysis. (**A**) Acetic acid content. (**B**) Isobutyric acid content. (**C**) Isovaleric acid content. (**D**) Butyric acid content. (**E**) Propionic acid content. (**F**) Valeric acid content. (**G**) Analysis of the correlation between biochemical indices in the colon of mice and the gut microbiota. (**H**) Analysis of the correlation between short chain fatty acids (SCFAs) in mice and the gut microbiota (positive correlation in red, negative correlation in blue). Data marked with different letters in each column represent significant differences (*p* < 0.05). Data marked with different letters in each column represent significant differences (*p* < 0.05).* *p* < 0.05, ** *p* < 0.01.

We performed a Spearman correlation analysis to investigate the relationship between the intestinal microbiota and short-chain fatty acids (SCFAs), as numerous studies have shown they are closely linked [37,38]. Our results similarly demonstrated that TKSO intervention increased SCFA levels in colitis mice. As shown in Figure 8A–F, analysis of the cecal contents revealed that TKSO treatment restored the levels of propionic acid, hexanoic acid, butyric acid, isobutyric acid, valeric acid, and isovaleric acid in DSS-challenged mice, with levels resembling those in the control group. Correlation analysis of colon biochemistry parameters indicated that *Turicibacter*, *Odoribacter*, *Desulfovibrio, Erysipelatoclostridium,* and *Parvibacter* were positively correlated with proinflammatory cytokines (IL-6, INF-β, and TNF-α) and negatively correlated with the expression of the tight junction proteins occludin and claudin-1 (*p* < 0.05) (Figure 8G). Conversely, *Turicibacter, Pseudomonas, Desulfovibrio,* and *Odoribacter* were negatively correlated with the six SCFAs (*p* < 0.05, *p* < 0.01), suggesting that an increased abundance of these bacteria may be a significant factor in triggering DSS. Furthermore, *Candidatus_Saccharimonas, Parvibacter, Norank_f_Muribaculaceae, Butyricoccus, Lachnospiraceae_NK4A136_group, Enterorhabdus, Bifidobacterium,* and *Lactobacillus* were positively correlated (*p* < 0.05) with tight junction proteins (occludin and claudin-1), and negatively correlated with inflammatory factors (IL-6, INF-β, and TNF-α). Figure 8H presents a correlation analysis between short-chain fatty acids (SCFAs) and specific gut microbiota, revealing a significant positive association between *Norank_f_Muribaculaceae*, *Butyricicoccus*, *Lachnospiraceae_NK4A136_group*, and *Lactobacillus* with the six SCFAs (*p* < 0.01, *p* < 0.001) [39]. SCFAs, the primary fermentation byproducts of dietary fiber in the gut, are pivotal in maintaining intestinal homeostasis and mitigating colitis by reinforcing the intestinal barrier and modulating immune responses [40,41]. Notably, butyric acid serves as a vital energy source for colonic epithelial cells, whereas acetic acid contributes to fat synthesis and gluconeogenesis [42,43]. Intriguingly, UC patients exhibit diminished levels of SCFA-producing bacteria, such as the aforementioned species, in the intestinal mucosa and cecal content compared to healthy controls. Our findings demonstrated that TKSO intervention resulted in a marked increase in SCFA-producing bacteria, especially Lactobacillus, which correlated with elevated levels of acetic, propionic, isobutyric, butyric, and valeric acids. As key butyrate producers, the increased abundance of *Lachnospiraceae_NK4A136_*group elevates butyrate levels within the gut, thereby enhancing intestinal homeostasis through promoting intestinal epithelial cell proliferation and barrier functions [44]. Consequently, the therapeutic potential of TKSO in alleviating DSS-induced colitis may be attributed to its capacity to modulate intestinal bacterial metabolism.

## 4. Conclusions

Our investigation affirms that the administration of TKSO reduces the severity of colitis induced by DSS and exerts a salutary impact on preserving the intestinal epithelial barrier integrity in mice afflicted with colitis. Specifically, TKSO therapy reinstated various physiological indices, manifesting as an elevation in body mass and colon length, alongside a decrease in the disease activity index (DAI) scores. Furthermore, TKSO supplementation mitigated the levels of proinflammatory cytokines, while augmenting the expression of tight junction proteins, thus fortifying intestinal integrity and enhancing epithelial barrier function. Additionally, TKSO actively modulated the gut microbiota’s composition and architecture, fostering the generation of short-chain fatty acids. These discoveries deepen our comprehension of TKSO’s role in mitigating intestinal inflammation and modulating immune-mediated disorders, ultimately guiding the progression and application of dietary lipid-based products. Despite the limitations of the article, the other findings and conclusions of this study still provide valuable references for the field.

## Figures and Tables

**Table 1:** The fatty acid composition and the relative content (%) of TKSO.

Composition	C16:0	C18:0	C18:1	C18:2	C18:3	C22:2
**Relative content (%)**	6.016	3.291	22.448	33.177	31.761	0.559
**Retain time (min)**	16.514	20.64	21.568	23.107	30.497	30.846

**Table 2:** Primer sequences used in the RT-qPCR assays in colonic tissue.

Gene	Primer Sequence	
	Forward Primer	Reverse Primer
IL-4	CATGGCGTCCCTTCTCCTGTG	GTTGTCATCCTGCTCTTCTTTCTCG
IL-6	GTGGTATCCTCTGTGAAGTCTCCTC	TTCTTGGGACTGATGCTGGTGAC
TNF-α	GTGGTTTGTGAGTGTGAGGGTCTG	CGCTCTTCTGTCTACTGAACTTCG
Occludin	CCTCTGACCTTGAGTGTGGATGAC	CCTCTGACCTTGAGTGTGGATGAC
Claudin-1	GTGTCCTACTTTCCTGCTCCTGTC	AGAAGGTGTTGGCTTGGGATAAGG
β-actin	TTGTAGAAGGTGTGGTGCCAGATC	GATGGTGGGAATGGGTCAGAAGG

## Data Availability

The original contributions presented in the study are included in the article/supplementary material, further inquiries can be directed to the corresponding authors.

## Supplementary Materials

The following supporting information can be downloaded at: https://www.mdpi.com/article/10.3390/foods13172668/s1, Table S1. The annealing temperatures of the relevant primer sequences. Fgure S1. The DSS drinking amount for each group of mice. Figure S2. The weight change rate for each group of mice.
