# Supplementary material for: Psidium guajava Seed Oil Reduces the Severity of Colitis Induced by Dextran Sulfate Sodium by Modulating the Intestinal Microbiota and Restoring the Intestinal Barrier
Source: Foods. 2024 Aug 24;13(17):2668. doi: 10.3390/foods13172668 (PMC11394160; doi:10.3390/foods13172668)

Table S1. The annealing temperatures of the relevant primer sequences.

Gene	Annealing temperature	
	**Forward Annealing temperature(℃)**	**Reverse Annealing temperature(℃)**
IL-4	57.8	61.3
IL-6	59.6	58.6
TNF-α	59.4	60.4
Occludin	59.7	59.9
Claudin-1	59.6	59.3
β-actin	60.4	59.1

Figure S1. The DSS drinking amount for each group of mice

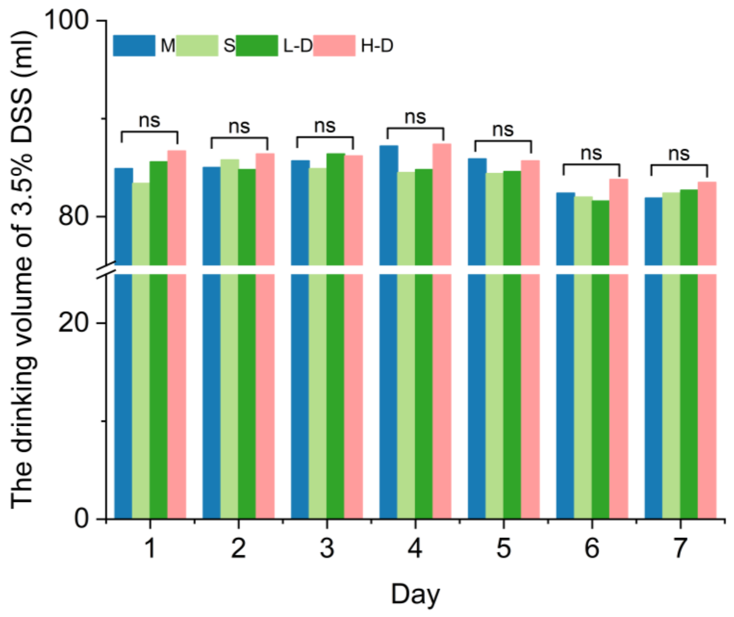

Figure S2. The weight change rate for each group of mice

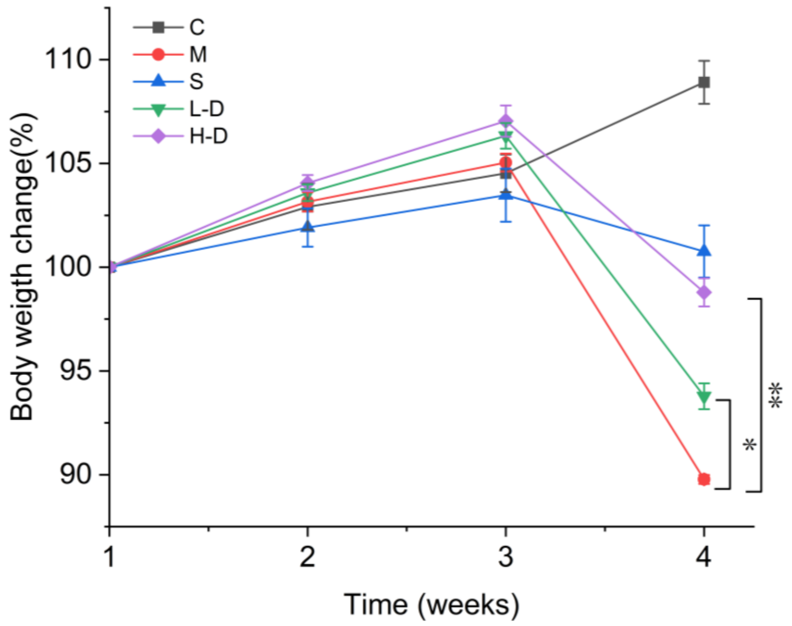

Supplement: Supplementary file 1 [file foods-13-02668-s001.zip › foods-3124948-supplementary.pdf]
